# Trends in Dialysis Industry Consolidation After Medicare Payment Reform, 2006-2016

**DOI:** 10.1001/jamahealthforum.2021.3626

**Published:** 2021-11-05

**Authors:** Caroline E. Sloan, Abby Hoffman, Matthew L. Maciejewski, Cynthia J. Coffman, Justin G. Trogdon, Virginia Wang

**Affiliations:** 1 Department of Medicine, Duke University Health System, Durham, North Carolina; 2 Health Services Research and Development Center of Innovation, Durham VA Health Care System, Durham, North Carolina; 3 Department of Health Policy and Management, Gillings School of Global Public Health, University of North Carolina at Chapel Hill, Chapel Hill, North Carolina; 4 Department of Population Health Sciences, Duke University School of Medicine, Durham, North Carolina; 5 Department of Biostatistics and Bioinformatics, Duke University School of Medicine, Durham, North Carolina

## Abstract

**Importance:**

The dialysis industry is highly concentrated, with large dialysis organizations now providing dialysis for more than 85% of patients with kidney failure in the United States. In 2011, Medicare introduced a new Prospective Payment System (PPS) for end-stage kidney disease, which bundled payment for dialysis care into 1 payment per patient. Trends in dialysis facility consolidation after the PPS went into effect are unknown.

**Objective:**

To determine whether the introduction of the PPS in 2011 was associated with an acceleration in acquisitions and closures of small dialysis chains (<20 facilities) and independently owned facilities.

**Design, Setting, and Participants:**

This retrospective cohort study included all Medicare-certified independent or small chain-affiliated dialysis facilities in the continental US between 2006 and 2016. Data were obtained from Medicare and the US Renal Data System and were analyzed in 2020.

**Exposures:**

The PPS.

**Main Outcomes and Measures:**

Discrete time hazard models were used to estimate the odds of acquisition and closure before the PPS (2006-2010) vs after the PPS (2011-2016). Analyses controlled for facility, market, and regional demographic characteristics. The average predicted marginal probabilities of acquisition and closure over time were estimated.

**Results:**

The proportion of small chain-affiliated and independently owned facilities declined from 29% (1383 of 4750 facilities) in 2006 to 15% (1038 of 6738) in 2016. Among 13 481 facility-years, 6352 (47%) were for profit, and mean (SD) census was 68 (59) patients. Overall, 3286 (24%) facilities opened during the observation period. The proportion of acquisitions that occurred each year varied from 1.1% (12 of 1065 facilities in 2015) to 7.2% (86 of 1192 facilities in 2012), while closures varied from 0.8% (9 of 1065 facilities in 2015) to 2.2% (28 of 1286 facilities in 2010), making both fairly rare. There was a 3.48 higher odds of acquisition in the post-PPS period compared with the pre-PPS period (95% CI, 1.62-7.47; *P* = .001). The odds of closure before and after the PPS were not statistically significantly different (odds ratio, 2.03; 95% CI, 0.61-6.73; *P* = .25). Facilities that opened during the observation period had a 7.2% higher predicted probability of acquisition compared with older facilities (95% CI, 5.4%-9.0%; *P* < .001).

**Conclusions and Relevance:**

In this cohort study of continental US Medicare-certified dialysis facilities, small-chain and independently owned facilities retained a declining share of the dialysis market. Further research should evaluate the effect of continued dialysis market consolidation on patient access, health care utilization, and clinical outcomes.

## Key Points

**Question:**

Was Medicare’s 2011 End-Stage Renal Disease Prospective Payment System (PPS) associated with dialysis facility acquisitions and/or closures?

**Findings:**

In this cohort study of 6738 Medicare-certified dialysis facilities in the continental US, small chain-affiliated and independently owned facilities had 3.48 higher odds of acquisition post-PPS (2011-2016) compared with pre-PPS (2006-2010). Risk of closure did not change.

**Meaning:**

Small-chain and independently owned facilities have retained a declining share of the dialysis market since Medicare payment reform, largely associated with acquisitions, rather than closures.

## Teaser

This cohort study aims to determine whether the introduction of the End Stage Renal Disease Prospective Payment System in 2011 was associated with an acceleration in acquisitions and closures of small dialysis chains and independently owned facilities.

## Introduction

Dialysis facilities in the United States currently treat more than 700 000 patients with end-stage kidney disease (ESKD).^1^ The dialysis industry has become highly concentrated over the past 2 decades, transitioning from an industry dominated by 5 large dialysis organizations (LDOs; ≥20 facilities) in the early 2000s to a duopoly dominated by 2 for-profit LDOs today.^2,3^ Patients receiving dialysis at facilities affiliated with for-profit LDOs have lower survival rates than patients receiving dialysis at smaller nonprofit chains and independent facilities.^4^ Dialysis facility acquisitions have been shown to have negative associations with clinical care and patient outcomes.^3,5^ Independent facilities that were acquired by LDOs in 2005, for instance, lowered their staff-to-patient ratios, reduced referrals for kidney transplant, and increased use of expensive injectable drugs.^3^ Hospitalizations for bacteremia and death increased after acquisition.^5,6,7^

Most US individuals with ESKD are covered by Medicare. In 2011, the Centers for Medicare & Medicaid Services (CMS) reformed its payment system for dialysis to reduce escalating spending in the Medicare ESKD program. Until 2011, Medicare reimbursed facilities for dialysis care in 2 parts: (1) a prospective payment for dialysis treatment regardless of modality, and (2) a separate, fee-for-service payment for drugs, laboratory tests, and supplies. Over time, the separately billable items for medications and diagnostic tests grew to comprise up to 40% of dialysis-associated costs.^8^ The 2011 Medicare ESKD Prospective Payment System (PPS) bundled the prior 2-component payment into a single prospective payment, adjusting for patient and facility characteristics.^9^ Under this system, dialysis facilities receive 1 payment per patient undergoing dialysis, regardless of the number of medications and laboratory tests administered during or after treatment.

In the run-up to the PPS, dialysis organizations, both large and small, warned of impending facility closures, decreased access to care, and increased consolidation.^9,10^ To date, studies have shown that the number of unique dialysis facilities actually increased after 2011,^11,12^ while facility closures declined.^12^ Most facilities have adapted to the PPS by instituting clinical and organizational changes such as lowering the doses of expensive injectable medications and modestly increasing the provision of historically less expensive dialysis modalities (eg, peritoneal dialysis).^8,11,13^ But the survival of small chains and independently owned dialysis facilities, which typically have fewer resources to withstand major financial shocks, has not been specifically evaluated in the post-PPS era. In this cohort study, we examine whether Medicare’s 2011 ESKD PPS was associated with an acceleration in acquisitions and closures of small dialysis chains and independently owned facilities from 2006 through 2016. We also identify facility and market factors associated with closure and acquisition during this time.

## Methods

### Study Design, Population, and Data Sources

We conducted a retrospective cohort study of Medicare-certified dialysis facilities at risk for acquisition or closure between 2006 and 2016. We defined “at-risk” as any facility that was either independent or part of a small chain (<20 facilities). To ensure that we included all at-risk facilities, even if they closed or were acquired in a given year, at-risk status was based on a facility’s status the year prior to the year in which the closure or acquisition outcome occurred. We excluded facilities that did not provide dialysis (eg, transplant only), were located in the noncontiguous US states (ie, Hawaii, Alaska), or were federally operated (eg, Department of Veterans Affairs).

Dialysis facility data are from the US Renal Data System, the national disease registry of patients with ESKD and ESKD-related facilities. Facility characteristics and operating statistics come from the Annual Facility Survey (form CMS-2744), which all Medicare-participating dialysis facilities must complete. To track facility closures and changes in ownership, we merged these data with the Medicare Provider of Service file, which contains information on facility addresses, chain affiliation, Medicare certification dates, and termination dates.^11,14,15,16^ The final sample included 2028 unique at-risk facilities from 2006 to 2016 (13 481 facility-year observations).

General population demographics are from the Area Health Resources Files. Demographics of patients with ESKD, including race and ethnicity, came from the Medical Evidence Report (form CMS-2728), which dialysis facilities complete for any new patient initiating treatment. These data were aggregated to generate market-level statistics for each year of the observation period. Markets were defined as Dartmouth Atlas hospital service areas (HSAs).^17^ There are 3436 HSAs nationwide, of which 1084 (32%) had 1 or more at-risk facilities during the observation period. This study was approved by the Duke University Health System Institutional Review Board and exempted from need of informed consent owing to use of secondary administrative and deidentified data.

### Measurement

The 2 primary dependent variables were acquisition and closure, each modeled separately. Acquisition was defined as change in ownership. All at-risk facilities were observed until they closed, were acquired by an LDO, or the observation time ended.

The primary independent variable of interest was the PPS indicator (ie, whether a given observation occurred before the PPS was implemented [2006-2010] or afterward [2011-2016]). We retained specific facility characteristics that are known to increase risk of organizational acquisition or closure: for-profit ownership similar to that of acquiring firms,^18^ firm size (eg, patient census, hemodialysis station occupancy),^18^ and newness (ie, recently opened facilities).^19,20^ We anticipated that at-risk facilities in more competitive (ie, less monopolistic) HSAs were more likely to close because they could not compete for patients with nearby chain-affiliated facilities.

The analysis controlled for other facility-level factors, including freestanding vs hospital based, small chain vs independent, rural vs urban, available dialysis modalities (in-center dialysis, home dialysis, or both), and proportion of patients enrolled in Medicare. We controlled for market-level factors (US region and facilities per HSA), regional demographics (per capita income, proportion of population living in urban areas, and ESKD incidence), and patient demographics (age, race, ethnicity, and employment status). Market competition was calculated using the Herfindahl-Hirschman Index (HHI). Index values range from 0 to 100, where a value of 0 reflects unconcentrated, competitive markets and values approaching 100 characterize concentrated, monopolistic markets. We treated all facilities within the same chain as a single entity.^19,21,22^ All facility-, market-, and region-level variables were lagged by 1 year because the associations of these variables with closure and acquisition were unlikely to be instantaneous.

### Statistical Analysis

The analysis presented here was conducted in 2020. We describe facility, market, and regional characteristics separately for facilities that were ever acquired, facilities that ever closed, and facilities that experienced neither outcome. We generated choropleth maps to illustrate the geographical distribution of acquisitions and closures by HSA throughout the US, using ArcGIS software, version 10.7 (Esri).^23^

We fit 2 discrete time hazard models separately to the acquisition and closure outcomes to estimate the odds that a facility was acquired or closed in any year between 2006 and 2016. The 2 outcomes were modeled separately because we hypothesized that covariate effects on acquisitions differed from covariate effects on closure. We focused on 3 main explanatory variables: (1) time in years, (2) post-PPS vs pre-PPS, and (3) the interaction between PPS and time. The interaction term assessed whether the annual rate of change in acquisitions or closures changed post-PPS compared with pre-PPS. Both models controlled for the facility and market characteristics previously described. To graphically illustrate trends in the annual probability of acquisition and closure, we estimated the average predicted marginal probabilities of acquisitions and closures over time, given that the outcome had not already been reached in an earlier year (eMethods in the Supplement). All statistical analyses were performed with Stata, version 16.0 (StataCorp). All tests were 2-sided, and *P* < .05 was considered statistically significant. This article follows the Strengthening the Reporting of Observational Studies in Epidemiology (STROBE) reporting guidelines for cohort studies.

## Results

### Descriptive Characteristics

**Figure 1.**
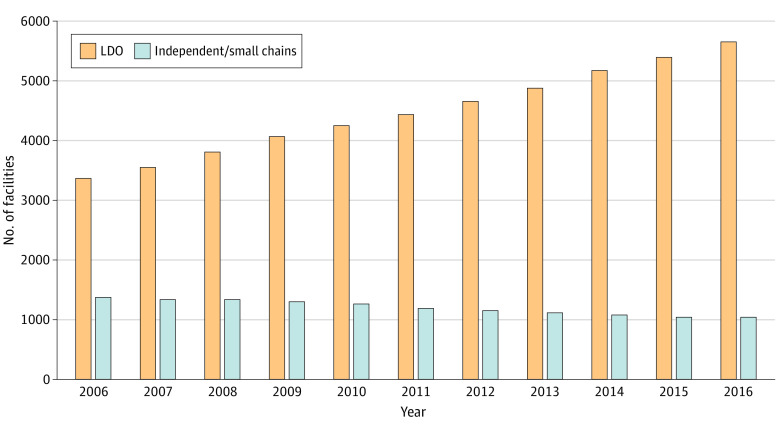
Number of Small Chain-Affiliated or Independently Owned Dialysis Facilities in the US, 2006-2016 The total number of unique dialysis facilities increased by 42% over the observation period, from 4750 in 2006 to 6738 in 2016. The absolute number of facilities associated with large dialysis organizations (LDOs) increased from 3367 in 2006 (71% of all facilities) to 5700 in 2016 (85%). The absolute number of at-risk facilities declined from 1383 in 2006 (29% of all facilities) to 1038 in 2016 (15%).

The total number of unique dialysis facilities increased by 42% between 2006 and 2016, from 4750 to 6738 (Figure 1). The number of facilities associated with LDOs increased by 69%, from 3367 in 2006 (71% of facilities) to 5700 in 2016 (85%). In contrast, the absolute number of at-risk facilities declined by 25%, from 1383 in 2006 (29% of facilities) to 1038 in 2016 (15%). The number of HSAs containing any at-risk facilities declined from 840 (24% of HSAs) in 2006 to 608 (18%) in 2016.

Among 13 481 at-risk facility-years in the sample, 6352 (47%) were for-profit, 5323 (40%) were affiliated with small chains (vs independently owned), and 9889 (73%) were in urban areas (Table). Mean (SD) census was 68 (59) patients and hemodialysis station occupancy was 53% (29%). Overall, 3286 (24%) facilities opened during the observation period. Mean (SD) HHI was 57 (31).

**Table.** Characteristics of Small Chain-Affiliated or Independently Owned Dialysis Facilities, Markets, and Regions From 2006-2016

Characteristic	No. (%)			
	Overall sample (n = 13 481)	Facilities that were ever acquired (n = 3211)^a^	Facilities that ever closed (n = 888)^a^	Facilities that neither were acquired nor closed (n = 9461)^a^
Dialysis facility				
For-profit ownership	6352 (47)	1755 (55)	421 (47)	4228 (45)
Freestanding facility (vs hospital based)	7778 (58)	1953 (61)	453 (51)	5422 (57)
Small chain (vs independent)	5323 (40)	1334 (42)	271 (31)	3763 (40)
Urban location	9889 (73)	2202 (69)	689 (78)	7057 (75)
No. of patients per facility, mean (SD)	68 (59)	77 (58)	43 (43)	67 (59)
% Hemodialysis station occupancy, mean (SD)^b^	53 (29)	59 (26)	44 (33)	52 (30)
Dialysis modalities offered				
In-center hemodialysis only	7475 (56)	1878 (59)	551 (63)	5111 (54)
In-center and home dialysis	5114 (38)	1239 (39)	242 (28)	3647 (39)
Home dialysis only	835 (6)	94 (3)	79 (9)	662 (7)
% Patients on Medicare, mean (SD)	90 (16)	93 (11)	88 (23)	89 (16)
New in observation period	3286 (24)	887 (28)	176 (20)	2238 (24)
General market (HSA) composition				
US region				
Northeast	2943 (22)	708 (22)	282 (32)	1971 (21)
Midwest	3928 (29)	1062 (33)	245 (28)	2644 (28)
South	4169 (31)	1004 (31)	256 (29)	2927 (31)
West	2441 (18)	437 (14)	105 (12)	1919 (20)
Total facilities per HSA, mean (SD)^c^	10 (15)	8 (15)	11 (14)	10 (15)
Dialysis market competition, mean HHI (SD)^d^	57 (31)	61 (32)	52 (29)	56 (30)
Regional demographics, mean (SD)				
Per-capita income, $1000	41 (13)	39 (13)	40 (13)	41 (13)
% Urban population	77 (25)	75 (25)	79 (25)	78 (25)
ESKD Incidence^e^	6 (7)	5 (4)	5 (4)	6 (8)
Patients with prevalent ESKD				
% Age <65 y	58 (7)	58 (8)	57 (8)	58 (7)
% White and non-Hispanic	56 (28)	60 (27)	57 (28)	55 (28)
% Employed	17 (6)	16 (5)	15 (5)	17 (6)

Abbreviations: ESKD, end-stage kidney disease; HHI, Herfindahl-Hirschman Index; HSA, hospital service area.

^a^ Facility-years for facilities that were ever acquired, facilities that ever closed, and facilities that were neither acquired nor closed do not add up to the facility-years overall because 20 facilities (equivalent to 79 facility-years) were acquired and then closed in a subsequent year.

^b^ Defined as total number of in-unit hemodialysis treatments provided divided by total number of possible hemodialysis treatment sessions. The number of possible treatment sessions is based on the number of hemodialysis stations in a facility and assumes an average of 3 shifts/d, 6 d/wk, 51 wk/y.

^c^ Includes facilities that were affiliated with large chains.

^d^ Facilities within a market under the same ownership (eg, in the same chain) were treated as a single firm.

^e^ ESKD incidence is defined as the number of patients with ESKD, per 10 000 general population in an HSA, per year.

### Acquisitions of Small Chain-Affiliated and Independently Owned Facilities

**Figure 2.**
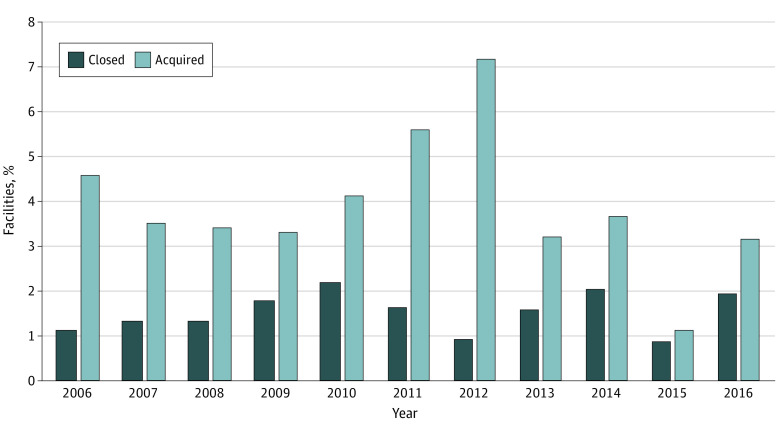
Observed Proportion of At-Risk Facilities That Were Acquired or Closed, 2006-2016 The proportion of facilities that were acquired each year varied between 1.1% and 7.2% during the observation period. The proportion of facilities that closed each year varied between 0.8% and 2.2%.

**Figure 3.**
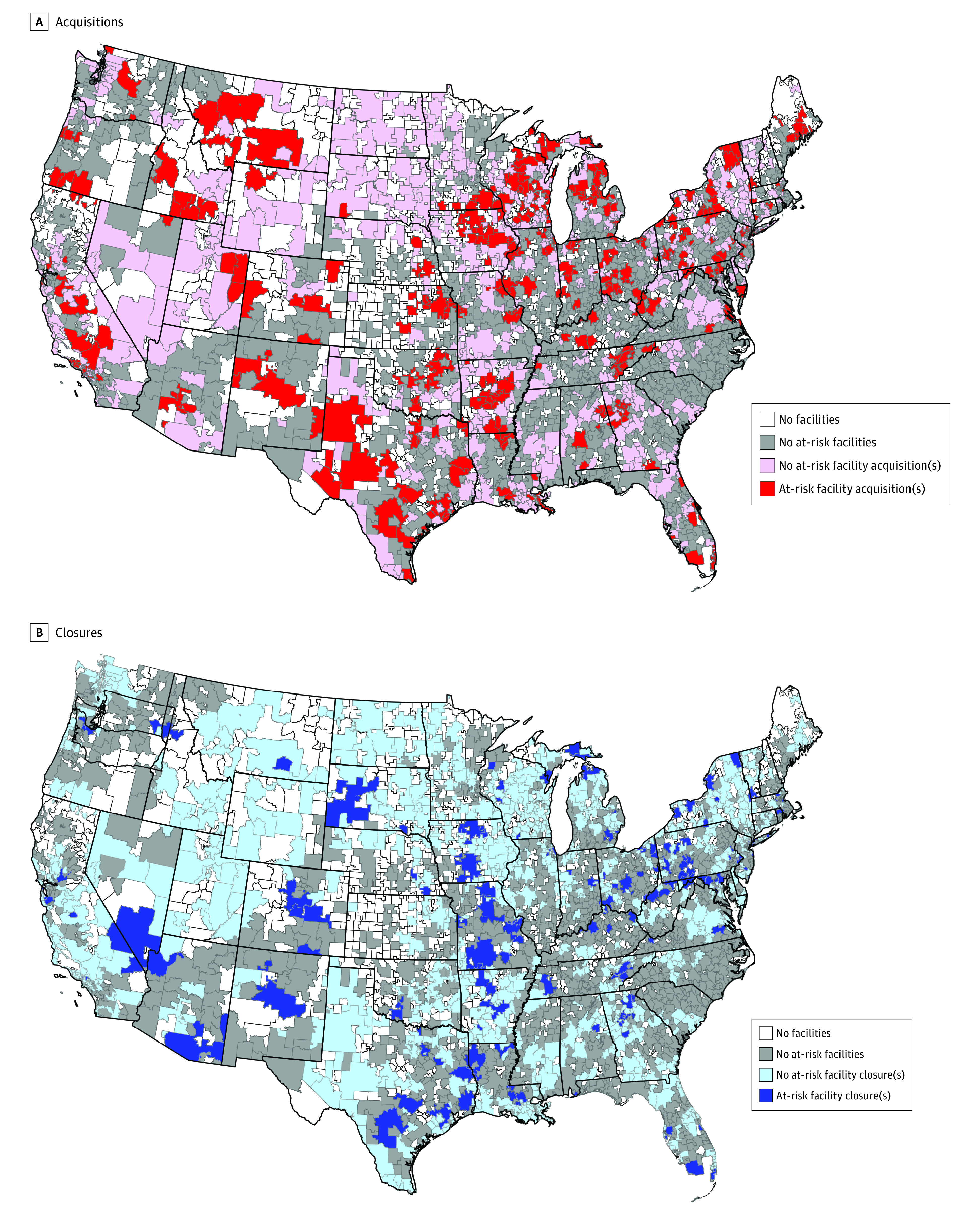
Proportion of At-Risk Facilities That Were Acquired and Closed in Each US Hospital Service Area, 2006-2016

Overall, 532 at-risk facilities were acquired from 2006 though 2016, equivalent to 26% of all facilities included in the cohort. The proportion of facilities that were acquired each year varied between 1.1% (12 of 1065) in 2015 and 7.2% (86 of 1192) in 2012 (Figure 2). Among the 3211 acquired facility-year observations, 1755 (55%) were for profit, mean (SD) annual patient census was 77 (58), mean (SD) annual hemodialysis station occupancy was 59% (26%), and 887 (28%) opened during the observation period (Table). Acquisitions were rare in states with few at-risk facilities (eg, South Carolina; Figure 3A). Most acquired facilities were located in urban locations (n = 2202 [69%]) and in the Midwest (n = 1062 [33%]) and the South (n = 1004 [31%]).

**Figure 4.**
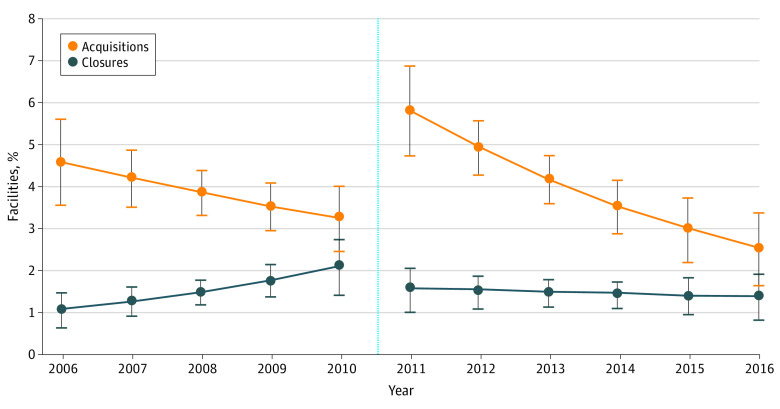
Adjusted Annual Predicted Probabilities of Acquisition and Closure Before and After Implementation of the Medicare End-Stage Renal Disease Prospective Payment System Error bars illustrate upper and lower bounds of 95% CIs. The light blue dotted line marks the year in which the Centers for Medicare & Medicaid Services began reimbursing facilities under the Prospective Payment System (2011).

In adjusted analysis, the odds of acquisition in the post-PPS period were 3.48 higher compared with the pre-PPS period (95% CI, 1.62-7.47; *P* = .001). The pattern of acquisitions fluctuated throughout the observation period. Before the PPS, the predicted probability of acquisition declined by 0.3% per year, but this decline was not statistically significant (95% CI, –0.7% to 0.03%; *P* = .07; Figure 4). The predicted probability of acquisition then increased statistically significantly in the year the PPS was implemented, from 3.2% (95% CI, 2.5%-4.0%) in 2010 to 5.8% (95% CI, 4.8%-6.9%; *P* < .001) in 2011. After this initial rise, the predicted probability of acquisition declined again, by 0.7% per year (95% CI, –1.1% to –0.3%; *P* < .001). The rates of decline in acquisitions in the pre-PPS and post-PPS periods were not statistically significantly different from each other (odds ratio [OR], 0.92; 95% CI, 0.80-1.05; *P* = .20).

Throughout the observation period, for-profit facilities had a 2.1% higher predicted probability of being acquired compared with nonprofit facilities (95% CI, 1.0%-3.2%; *P* < .001; eTable in the Supplement). For each additional 10 patients in a facility’s census, there was a small but statistically significant increase in the probability of acquisition (0.2%; 95% CI, 0.1%-0.2%; *P* < .001). For each additional 10% in hemodialysis station occupancy, there was a 0.4% increase in the probability of acquisition (95% CI, 0.3%-0.6%; *P* < .001). Facilities that opened during the observation period had a 7.2% higher predicted probability of acquisition compared with older facilities (95% CI, 5.4%-9.0%; *P* < .001).

### Closures of Small Chain-Affiliated and Independently Owned Facilities

Ten percent (n = 204) of at-risk facilities closed during the observation period. The proportion of facilities that closed each year varied from 0.8% (9 of 1065) in 2015 to 2.2% (28 of 1286) in 2010 (Figure 2). Among the 888 facility-year observations of closure, 421 (47%) were for profit, mean (SD) annual patient census was 43 (43), mean (SD) annual hemodialysis station occupancy was 44% (33%), and 176 (20%) opened during the observation period. A total of 689 (78%) closures occurred in urban areas. Although only 2943 of 13 481 (22%) facility-years were located in the Northeast, 282 of 888 (32%) closed facilities were located in that region (Table). Closures were rare in states that had few at-risk facilities (Figure 3B).

In adjusted results, the odds of closure pre-PPS and post-PPS were not statistically significantly different (OR, 2.03; 95% CI, 0.61-6.73; *P* = .25). In the pre-PPS period, the annual probability of closure increased by 0.3% per year (95% CI, 0.04%-0.5%; *P* = .02; Figure 4). The predicted probability of closure declined from 2.1% (95% CI, 1.4%-2.7%) to 1.5% (95% CI, 1.0%-2.1%) the year the PPS was implemented, but this decline was not statistically significant (*P* = .20). In the post-PPS period, the probability of closure remained stable at 1.4% to 1.5% per year (decline in predicted closure probability per year, 0.03%; 95% CI –0.2% to 0.2%; *P* = .72). The annual change in closure rates was statistically significantly lower in the post-PPS period compared with the pre-PPS period (OR, 0.82; 95% CI, 0.67-0.99; *P* = .04).

Higher patient census was associated with a reduced probability of closure (per 10 additional patients, −0.3%; 95% CI –0.4% to –0.1%; *P* < .001), and higher market concentration was associated with lower probability of closure (per a 10-point increase in HHI, −0.1%; 95% CI –0.2% to –0.02%; *P* = .02). Contrary to the study hypotheses, facility newness and hemodialysis station occupancy were not associated with closures. See eTable in the Supplement for full regression results.

## Discussion

We evaluated the association between Medicare’s ESKD PPS and acquisitions and/or closures of small chain-affiliated and independently owned dialysis facilities. Overall, acquisitions and closures were fairly rare.

The probability of acquisition increased considerably immediately after the PPS, then declined from 2012 through 2016. The increase in acquisitions of at-risk facilities immediately after the PPS may be attributed to the financial uncertainties that dialysis facilities anticipated in the years leading up to payment reform.^24^ With conversion from a partial prospective payment to a fully comprehensive, bundled payment for dialysis treatment, these facilities could no longer rely on fee-for-service payments to maintain revenues. By merging with LDOs, newly acquired facilities may have benefited from bulk discounts on medications and supplies, centralized laboratories, and lower per-patient fees for electronic medical records,^19,25^ generating economies of scale that increased their likelihood of operability and survival.^26^ Once revenue margins proved to be stable in the post-PPS period,^27^ at-risk facilities may have seen less of a need to merge with LDOs, so acquisitions declined.

Facility closures stabilized in the post-PPS era, consistent with a recent evaluation of closures among all dialysis facilities (including LDOs) between 2006 and 2015.^12^ The low rate of at-risk facility closures since 2011 is likely related to a combination of factors. First, Medicare PPS payment rates were intended to remain budget neutral. That is, facilities that were paid a PPS rate received roughly the same payment as they had received under the fee-for-service system, minus a 2% reduction to adjust for assumed efficiencies that would result from the PPS. In fact, in the years following the PPS, the use of erythropoiesis-stimulating agents (ESAs) declined so much that reimbursement rates were reduced by another 12% in 2014.^10^ Second, Medicare payments were adjusted for patient case mix, facility size, and geographic location, so facilities caring for sicker patients received higher reimbursements. Third, the population with ESKD grew 43% between 2006 and 2016, from 506 764 to 726 331.^1^ The overall number of dialysis facilities in the United States correspondingly grew by 42%. With this increased demand for dialysis, the dialysis industry will likely continue growing, and closures are likely to remain stable in the near future.

Moving forward, it will be important for policy makers to monitor the dialysis industry closely, because consolidation can have important effects on the ability of patients with ESKD to access and use health care, as well as on their outcomes.^3,4,5,6,7^ With only 27% of at-risk dialysis facilities located in rural areas, for example, even small numbers of acquisitions and closures could have important effects on rural patients’ choice of facility. The literature on consolidation in the hospital industry has shown that when health systems merge, their market power increases and they are able to leverage this into higher reimbursement rates with private insurers.^28,29^ In contrast, when dialysis facilities merge, their negotiating power increases only modestly because the majority of patients with ESKD are covered by traditional Medicare.^30^ However, LDOs can use a number of other strategies to increase their revenue. First, they can favor patients covered by private insurance plans, which reimburse more generously than Medicare, as we recently showed.^30^ This strategy could increase disparities in dialysis facility choice between privately and publicly insured patients. Second, facilities can reduce their operating costs by lowering their staff-to-patient ratios or by hiring staff who are less skilled and therefore take lower salaries,^3^ leading to worse outcomes for patients.^3,6^ Facilities may also reduce their use of ESAs and instead treat anemia with red blood cell transfusions, which are reimbursed separately from the PPS.^31^ Chronic kidney disease treatment guidelines recommend avoiding transfusions whenever possible,^32,33^ because transfusions can sensitize patients to human leukocyte antigens and reduce opportunities for kidney transplant.^34^ Third, dialysis facilities can attempt to maintain their client base by limiting referrals for kidney transplants, as shown previously.^3^ While any facility may pursue these strategies, LDOs have pursued them more frequently than small chains and independent facilities.^3,6,25,30,31^ These strategies all have the potential to reduce access to quality care and worsen clinical outcomes.^3,5,6,7,12^

Policy makers should be aware of the facility- and market-level characteristics that are most strongly associated with acquisitions and closures. Market size and HHI were not associated with acquisitions and had minimal association with closures. In contrast, several individual facility characteristics were associated with acquisitions and closures. Smaller at-risk facilities were more likely to close, while newer, larger, and potentially more profitable dialysis facilities (ie, higher hemodialysis station occupancy) were more likely to be acquired. These findings suggest that LDOs are increasing their share not only of dialysis facilities nationwide, but also of patients undergoing dialysis. Given the negative association that ownership by LDOs has been shown to have with clinical outcomes,^3,4,6,7,25,35^ policy makers should take measures to protect the small chain-affiliated and independent facilities at highest risk of acquisition or closure.

### Limitations

Study limitations should be noted. First, other major health policies were implemented around the same time as the PPS, including the Affordable Care Act and other dialysis-focused initiatives such as changes in ESA labeling^36^ and the ESKD Quality Incentive Program.^37^ These policies may have had simultaneous effects on acquisitions and closures, although these effects were likely modest. The ESKD Quality Incentive Plan in particular has not yet led to improved quality of care, perhaps owing to the large number of included quality measures, frequent changes in quality measures year to year, and the low reimbursement penalty (maximum, 2%) associated with low scores.^38,39^ Second, we omitted variables with substantial missing or unreliable data (eg, staff-to-patient ratio) from the analysis. These variables may have influenced facilities in their decision to be acquired or close. Third, we did not have data on facilities’ financial performance or the extent of private insurance coverage (vs Medicare or Medicaid) and therefore could not assess the association between these characteristics and the risk of acquisition or closure. Fourth, we cannot completely exclude the possibility of competing risk: some closures may have prevented the observation of an acquisition and vice versa. However, the profiles of acquired facilities were different from the profiles of facilities that closed, so competing risk is unlikely to have affected the results. Finally, despite all attempts to adjust for factors to minimize bias, unmeasured confounders may exist and causality cannot be proven.

## Conclusions

This cohort study shows that while there was a spike in dialysis facility acquisitions in the immediate aftermath of the PPS, the overall trend in acquisitions and closures has been one of decline. Despite this, the share of the dialysis market that comprises small dialysis chains and independent facilities is shrinking. Further research should evaluate the effect of continued dialysis market consolidation on patient access, health care utilization, and clinical outcomes.

## Notes

Supplement. **eMethods.** Statistical analysis **eTable.** Logistic regression results: Predictors of dialysis facility acquisition and closure, 2006-2016
