# Supplementary material for: Trends in Dialysis Industry Consolidation After Medicare Payment Reform, 2006-2016
Source: JAMA Health Forum. 2021 Nov 5;2(11):e213626. doi: 10.1001/jamahealthforum.2021.3626 (PMC8796909; doi:10.1001/jamahealthforum.2021.3626)
Supplement: Supplement. — eMethods. Statistical analysis eTable. Logistic regression results: Predictors of dialysis facility acquisition and closure, 2006-2016 [file jamahealthforum-e213626-s001.pdf]

Supplemental Online Content

Sloan CE, Hoffman A, Maciejewski ML, Coffman CJ, Trogdon JG, Wang V. Trends in dialysis industry consolidation after Medicare payment reform, 2006-2016. *JAMA Health Forum*. 2021;2(11):e213626. doi:10.1001/jamahealthforum.2021.3626

eMethods. Statistical analysis
eTable. Logistic regression results: Predictors of dialysis facility acquisition and closure, 2006-2016

This supplemental material has been provided by the authors to give readers additional information about their work.

© 2021 Sloan CE et al. *JAMA Health Forum.*

eMethods. Statistical analysis

We fit two discrete time hazard models separately to the acquisition and closure outcomes, to predict the odds that a facility was acquired or closed in any year between 2006 and 2016, conditional that the outcome did not occur in prior years. The two outcomes were modeled separately because we hypothesized that predictors of acquisition differed from predictors of closure. Outcomes were modeled as follows, using logistic regression and robust standard errors:

$$\log\left(\frac{p_{ij}}{1-p_{ij}}\right) = \beta_{1j}(t) + \beta_2(PPS) + \beta_3(t * PPS) + \beta_4\boldsymbol{C'}_{\boldsymbol{i}} + \beta_5\boldsymbol{D'}_{\boldsymbol{ij}}$$

where p is the probability of the observed outcome (acquisition or closure), i denotes each "at-risk" dialysis facility, j denotes year, and t is a continuous variable representing time in years. We focused on three main explanatory variables: β_1 represents the effect of time on the odds of acquisition/closure; β_2 evaluates whether the odds of acquisition/closure were different in the post-PPS era compared to the pre-PPS era; and β_3, the interaction between the PPS variable and time, assesses whether the annual rate of change in acquisitions/closures changed in the post-PPS era compared to the pre-PPS era. The vector $\boldsymbol{C_i}$ represents time-invariant variables, which remain constant for each facility in each year (e.g. US region). The vector $\boldsymbol{D_{ij}}$ represents time-varying variables, which can take on different values in different years (e.g. hemodialysis station occupancy). The unit of observation is the facility-year, with up to a maximum of J observations per facility. The first observation for a facility was 2006, or the year it first opened if it opened after 2006. We used a p-value of 0.05 as a measure of statistical significance. All statistical analyses were performed with Stata software, version 16.0 (StataCorp).

Each facility was at risk for the outcome in any given year as long as it was still in our sample. In the analysis of acquisitions, facilities that closed were censored from the analysis on the date of closure. In the analysis of closures, facilities that were acquired by large dialysis

© 2021 Sloan CE et al. *JAMA Health Forum.*

organizations (LDOs) were considered to no longer be “at risk” and thus were censored from the analysis on the date of acquisition. Facilities that were acquired by small chains were considered to remain at risk for closure and therefore were not censored.

To graphically illustrate trends in the annual probability of acquisition and closure, we plotted the average predicted marginal probabilities of acquisitions and closures over time, given that the outcome had not already been reached in an earlier year. The methods described here are similar to those used for previously published discrete time hazard models (1,2).

eReferences

1. Calcagno JC, Crosta P, Bailey T, Jenkins D. Does Age of Entrance Affect Community College Completion Probabilities? Evidence from a Discrete-Time Hazard Model. *Educ Eval Policy Anal*. 2007;29(3):218-235.

2. Pozniak AS, Hirth RA, Banaszak-Holl J, Wheeler JRC. Predictors of Chain Acquisition among Independent Dialysis Facilities. *Health Serv Res*. 2010;45(2):476-496. doi:10.1111/j.1475-6773.2010.01081.x

© 2021 Sloan CE et al. *JAMA Health Forum.*

eTable. Logistic regression results: Predictors of dialysis facility acquisition and closure, 2006-2016

	Acquisitions				Closures			
	OR	LCI	UCI	p-value	OR	LCI	UCI	p-value
**Year**	0.91	0.83	1.01	0.07	1.19	1.03	1.38	0.02
**PPS**	3.48	1.62	7.47	<0.01	2.03	0.61	6.73	0.25
**Years since PPS** [a]	0.92	0.80	1.05	0.20	0.82	0.67	0.99	0.04
**Dialysis facility characteristics**								
Previously acquired in observation period	--	--	--	--	0.91	0.47	1.76	0.78
For profit ownership	1.73	1.31	2.29	<0.001	1.28	0.77	2.14	0.34
Freestanding facility [b]	0.69	0.52	0.92	0.01	1.08	0.61	1.89	0.80
Small chain (vs independent)	0.98	0.80	1.20	0.85	0.92	0.67	1.27	0.62
Urban location	0.73	0.53	1.01	0.06	1.30	0.77	2.20	0.33
Number of patients per facility	1.00	1.00	1.01	<0.001	0.98	0.97	0.99	<0.001
Hemodialysis station occupancy	1.01	1.01	1.02	<0.001	1.00	0.99	1.00	0.33
Dialysis modalities offered								
In-center hemodialysis only	--	--	--	--				
In-center and home dialysis	0.98	0.79	1.20	0.81	0.71	0.49	1.02	0.06
Home dialysis only	0.63	0.39	1.01	0.05	0.70	0.37	1.32	0.27
Proportion of patients currently enrolled in Medicare	1.02	1.00	1.03	0.01	0.99	0.98	1.00	<0.01
New in observation period	3.99	3.07	5.20	<0.001	0.72	0.49	1.05	0.08
**General market (HSA) composition**								
US region								
Northeast, %	--	--	--	--				
Midwest, %	1.60	1.21	2.13	<0.001	0.49	0.32	0.75	<0.01
South, %	1.68	1.23	2.28	<0.001	0.57	0.37	0.88	0.01
West, %	1.09	0.78	1.52	0.63	0.53	0.30	0.95	0.03

© 2021 Sloan CE et al. *JAMA Health Forum.*

Total facilities per HSA	1.00	1.00	1.01	0.40		1.01	1.00	1.02	0.01
Dialysis market competition (HHI) [c]	1.00	1.00	1.01	0.20		0.99	0.98	1.00	0.02
**Regional characteristics**									
Per capita income	1.01	1.00	1.02	0.24		1.00	0.99	1.01	0.89
% urban population	1.01	1.00	1.01	0.05		0.99	0.98	1.00	0.20
ESRD incidence [d]	0.99	0.97	1.01	0.43		1.00	0.98	1.02	0.79
% age <65 years	1.02	1.01	1.04	0.01		0.97	0.95	1.00	0.02
% White and non-Hispanic	1.01	1.01	1.02	<0.001		1.00	0.99	1.01	0.72
% employed	0.97	0.95	0.99	<0.01		0.96	0.93	0.99	0.02

LCI: lower confidence interval; UCI: upper confidence interval; HSA: hospital service area; HHI: Herfindahl-Hirschman Index of dialysis market concentration; PPS: prospective payment system; OR: odds ratio.

[a] Interaction between the PPS variable and time.

[b] vs. hospital-based

[c] Facilities within a market under the same ownership (e.g., in the same chain) were treated as a single firm.

[d] ESRD incidence is defined as the number of new ESRD patients per 10,000 general population in HRR, per year.

© 2021 Sloan CE et al. *JAMA Health Forum.*
